# Can the DSE Fungus *Exserohilum rostratum* Mitigate the Effect of Salinity on the Grass *Chloris gayana*?

**DOI:** 10.3390/plants14162537

**Published:** 2025-08-15

**Authors:** Natalia Elizabeth Tobar Gomez, Marcos Ameijeiras, Hernan E. Benitez, Federico N. Spagnoletti, Viviana M. Chiocchio, Raúl S. Lavado

**Affiliations:** 1 Facultad de Agronomía, Universidad de Buenos Aires, Buenos Aires C1417DSE, Argentinalavado@agro.uba.ar (R.S.L.); 2 Instituto de Investigaciones en Biociencias Agrícolas y Ambientales (INBA), Facultad de Agronomía, Universidad de Buenos Aires, Buenos Aires C1417DSE, Argentina; 3 Instituto de Tecnología (INTEC), Universidad Argentina de la Empresa (UADE), Buenos Aires, Argentina

**Keywords:** salt-affected soils, dark septate endophytes, salt tolerance, K/Na ratio

## Abstract

Dark septate endophytes (DSEs) are commonly found in saline environments, such as the Flooding Pampas (Argentina), where the forage grass *Chloris gayana* has been introduced. This study evaluated the effect of salinity on the DSE fungus *Exserohilum rostratum*, isolated from *C. gayana*, and its contribution to the grass’s salinity tolerance. Two greenhouse experiments were conducted under three salinity levels (0, 40, and 80 meq Na·L^−1^), with and without fungal inoculation. Fungal growth, root colonization, functional traits, plant biomass, chemical composition, and salinity tolerance indices were assessed. The fungus tolerated salinity and colonized roots, showing qualitative evidence of enzyme production and phosphate solubilization. In both experiments, shoot and root biomass decreased with increasing salinity. Inoculation significantly enhanced shoot biomass only under non-saline conditions in the first experiment, whereas in the second experiment no inoculation effect was observed on shoots. For roots, no effect of inoculation occurred in the first experiment, but a positive interaction between salinity and inoculation was recorded in the second experiment, where moderate salinity increased root biomass in inoculated plants. The K/Na and Ca/Na ratios decreased under salinity regardless of inoculation, indicating limited influence on ionic balance. These results suggest that although *E. rostratum* tolerates salinity and expresses functional traits, its ability to enhance plant performance under stress is context-dependent and restricted to specific conditions.

## 1. Introduction

Vascular plants host a diverse range of fungal species in their roots, establishing various types of relationships and symbiotic interactions [1]. Among these, dark septate endophytes (DSEs) are particularly notable and frequently found in extreme environments, including saline soils [1,2,3]. Soil salinity is one of the major environmental constraints affecting both microbial communities and higher plants. It influences numerous morphological, physiological, biochemical, and molecular traits, primarily through osmotic stress, ion toxicity, and nutrient imbalances [4]. Salinity has been reported to reduce soil enzyme activities [5] although the effects of salinity on enzymes is still not fully understood. Salinity also decreases fungal colonization rates [6]. While research on the effects of salinity on fungi dates back to studies on human pathogens a century ago [7], investigations on agriculturally relevant fungi are relatively recent and limited [8].

Several studies indicate that plant tolerance to saline environments can be enhanced through symbiosis with DSE fungi [3,9,10]. This increased tolerance is largely attributed to improved water and nutrient uptake, facilitated by fungal colonization [11]. DSE fungi, like other soil microorganisms, play a critical role in increasing inorganic phosphorus (P) availability in saline soils [12,13] and mineralizing organic compounds in the rhizosphere [14].

Saline environments occur across a wide range of climatic regions, including temperate-humid areas such as the “Flooding Pampas” of central Argentina, where approximately 60% of soils are affected by hydrohalomorphic processes [15]. In the Flooding Pampas region, soil salinity is mainly dominated by sodium chloride salts, which is the primary component responsible for saline stress in local agricultural systems [15]. In recent years, *Chloris gayana* Kunth (known as Rhodes grass), a subtropical C4 perennial grass, native to southern and eastern Africa, has been successfully cultivated in these saline soils due to its drought and salt tolerance [16,17,18]. Salt tolerance in plants is classically defined as the ability to maintain growth and productivity under saline conditions. On the other hand, seed germination is usually affected by salinity [19], while the sensitivity in the germination stage indicates that *C. gayana* can be considered tolerant to salts [20]. The salt tolerance index (STI) is positively correlated with potential yield under salt stress [21]. One direct effect of salinity, when having crossed a threshold is the tendency to decrease chlorophyll content in several species [22].

Plants employ various mechanisms to mitigate salt stress. One strategy involves increasing foliar water content in order to dilute or sequestrate salts at sublethal concentrations (leaf succulence) [23,24]. Other mechanisms are to exclude Na^+^ entering roots, namely the compartmentalizing Na^+^ into the vacuole or sequestered into older leaves which will be dropped [25]. Oi et al. [26] indicates that *C. gayana* accumulates high Na^+^ concentrations in its shoots, but actively secretes salt crystals via specialized salt glands located on its leaves [26].

Non-tolerant plants suffer from ionic imbalance and exhibit K-Na antagonism in saline soils. Conversely, the salt-tolerant plants have developed mechanisms maintaining a proper K^+^/Na^+^ ratio for growth in salt-affected soils [25]. A crucial determinant of salinity tolerance in plants is not only the maintenance of an optimal potassium (K)/sodium (Na) ratio but also calcium (Ca)/Na ratios as well as magnesium, iron, etc. [19,25]. The mechanisms for Na exclusion and/or preferential K uptake help sustain a high K/Na ratio [26,27]. Additionally, selective K uptake, reflected in the relationship between the plant K^+^-Na^+^ relation and the soil K^+^/Na^+^ ratio, plays a critical role in salt tolerance [28,29].

This study aimed to evaluate the salt tolerance of the fungus *Exserohilum rostratum*, a DSE fungus isolated from the roots of the grass *Chloris gayana*, as well as the grass’s own tolerance to salinity. We also investigated the impact of their symbiotic interaction on plant performance and chemical composition under saline conditions in a greenhouse experiment.

## 2. Material and Methods

### 2.1. Isolation of DSE Fungi from Chloris Gayana Roots and Taxonomic Identification

A DSE fungus was isolated from the roots of *C. gayana* (cv. Finecut) growing in the Punta Indio District, Argentina (35°24′14.51″ W, 57°43′24.98″ S). The fungal isolate was identified as *Exserohilum rostratum*, and its DNA sequences were deposited in GenBank. A detailed description of the identification procedure is available [30]. To confirm its endophytic nature, a reinfection assay was performed following Koch’s postulates.

### 2.2. Fungal Characterization

#### 2.2.1. Salinity Tolerance

The fungus was cultured on malt extract agar (AEM), and inoculum was obtained from actively growing colonies. Mycelial discs were transferred to Erlenmeyer flasks containing malt broth supplemented with NaCl at 10 and 100 meq Na/L. A control treatment (without salt) was also included. The cultures were incubated in an orbital shaker under controlled conditions. After incubation, fungal biomass was harvested, dried in an oven until a constant weight was reached, and the Na^+^ concentration in the mycelium was quantified using standard techniques [31].

#### 2.2.2. Enzyme Activity

The qualitative enzymatic production in salinized AEM was performed: cellulase using carboxymethyl cellulose, amylase using soluble starch from potato, and lignin peroxidase using Azure-B medium was assessed, following the methodology described by Levin et al. [32].

#### 2.2.3. Medium Acidification Capacity

The acidification capacity of the fungal culture medium was evaluated following the methodology of Pandey et al. [33]. Fungal cultures were incubated for 12 days under agitation, centrifuged at 5000 rpm (Jouan centrifuge, Br4i model, Jouan SAS, Saint-Herblain, France), and the supernatant was filtered. The pH of the supernatant was measured using a pH meter (Conductronic PC45, Conductronic Instruments, Barcelona, Spain).

#### 2.2.4. Phosphate Solubilization Capacity

The ability of the strain to solubilize phosphorus in saline substrates was assessed using tricalcium phosphate as the sole P source. Inoculated cultures were incubated for 6 days at 25 °C in the dark in a nutrient medium. After incubation, both the halo diameter and colony diameter were measured to evaluate phosphate solubilization efficiency [34].

#### 2.2.5. Root Colonization

**Figure 1:**
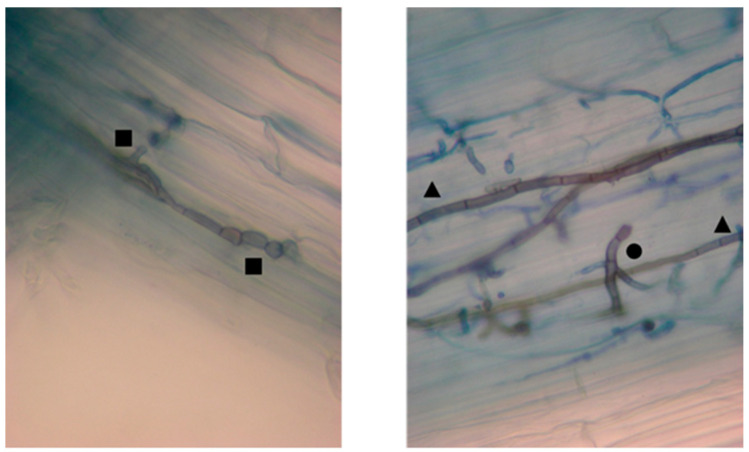
Photographs from trypan blue-stained roots under stereoscopic microscope (400×) showing *C. gayana* root colonization. Symbols: black squares denote the presence of *E. rostratum* intraradical septate hyphae, triangles denote *E. rostratum* extraradical septate hyphae, and circles denote an entry point.

The percentage of root colonization by the fungal isolate was determined through microscopic observation following the methodology of Barrow and Aaltonen [35]. Figure 1 records the endophytic character of the fungi.

### 2.3. Greenhouse Experiments and Plant Analyses

Two independent experiments were conducted in a greenhouse at the School of Agronomy, University of Buenos Aires, Argentina. In both cases, 1.5 L pots were used, filled with a substrate composed of dried and sieved soil (collected from the same site as the fungal isolate), sand, and perlite (1:1:1). The soil used in this study was classified as a Typic Natraqualf (US soil taxonomy) and presented the following physicochemical properties: silty clay loam texture at the surface (25% clay, 42.5% silt, and 32.5% sand), organic matter content of 2.3 ± 0.17%, available phosphorus of 4.5 ppm, pH (paste) of 9.2 ± 0.03, and electrical conductivity of 2.16 ± 0.07 dS·m^−1^ (mean ± standard error). However, other sampling data indicated higher EC values, reaching around 8 dS m^−1^.

The experimental design included six treatments: three salinity levels: N0: irrigation with distilled water (control); N40: irrigation with a NaCl solution containing 40 meq Na·L^−1^; and N80: irrigation with a NaCl solution containing 80 meq Na·L^−1^. Each salinity level was tested with and without inoculation with the DSE fungus *Exserohilum rostratum*, resulting in the following treatments: N0, N40, N80, N0 + DSE, N40 + DSE, and N80 + DSE. There were six replicates per treatment.

Pots were irrigated to maintain soil moisture close to field capacity, preventing water stress interference. Substrate salinity was maintained at approximately 0.2, 4, and 8 dS/m for N0, N40, and N80 treatments, respectively, throughout the 90-day experimental period.

Plants were fertilized with nitrogen (N) every two weeks, starting 30 days after sowing, using a solution of 0.5 g ammonium nitrate/L of water. The pots received an initial dose of NPK fertilizer in solution.

#### 2.3.1. Fungal Inoculation

The fungal inoculum was prepared from actively growing *Exserohilum rostratum* colonies on AEM [36]. The inoculum was applied to a sterile soil–perlite–sand (1:1:1) mixture, saturated with a 0.2% malt extract broth solution. This fungal inoculum was mixed with the potting substrate at a 3% (v/v) ratio before sowing the host plants.

#### 2.3.2. Plant Growth and Physiological Measurements

At harvest, aerial and root biomass were dried at 65 °C until a constant weight was reached in both experiments. The salinity tolerance index (STI) was calculated based on biomass reduction under extreme salinity conditions (N80 vs. N0) for both inoculated and non-inoculated plants, where STI = dry weight of salt-treated plants (N80)/dry weight of control (N0) [19,21].

In the first experiment, leaf N and P concentrations were determined using standard Kjeldahl and total P analysis techniques [37]; crude protein (CP) content and in vitro forage digestibility (acid-detergent fiber) content (FD), were determined [38].

In the second experiment, Na, K, and Ca concentrations in plant roots and leaves were measured using atomic absorption spectrophotometry after wet digestion with nitric and perchloric acids. The same cations were quantified in the substrate from the saturation extract [31]. K/Na and Ca/Na ratios and K and Ca selectivity were calculated. Leaf succulence was estimated as the ratio of fresh leaf biomass to dry biomass [23]. The leaf greenness index, a non-destructive, portable method to assess the chlorophyll content in leaves, was determined (SPAD-502, Minolta Corp., Ramsey, NJ, USA). Fifteen determinations per pot were recorded.

### 2.4. Statistical Analysis

Fungal colonization frequency was determined [39]. The data for aerial biomass, root biomass, foliar N, P, Na, K, crude protein, digestibility, and salinity tolerance indices were analyzed using ANOVA in RStudio (version 1.1.453 for Windows; RStudio Team, 2015) to assess the effects of the experimental factors applied. Data normality and homogeneity of variance were tested. When significant treatment effects were detected, Tukey’s post hoc test (*p* < 0.05) was applied for multiple comparisons.

## 3. Results

### 3.1. Fungus Response to Salinity

Table 1 shows that the mycelium biomass of *E. rostratum* increased significantly in the solution containing 10 meq Na/L, compared to the control, but the biomass reverted to the control value in the solution containing 100 meq Na/L. The concentration of Na in the dry mycelium was low in the control and the 10 meq Na/L concentration but increased abruptly and significatively when the fungus was subjected to a concentration of 100 meq Na/L.

**Figure 2:**
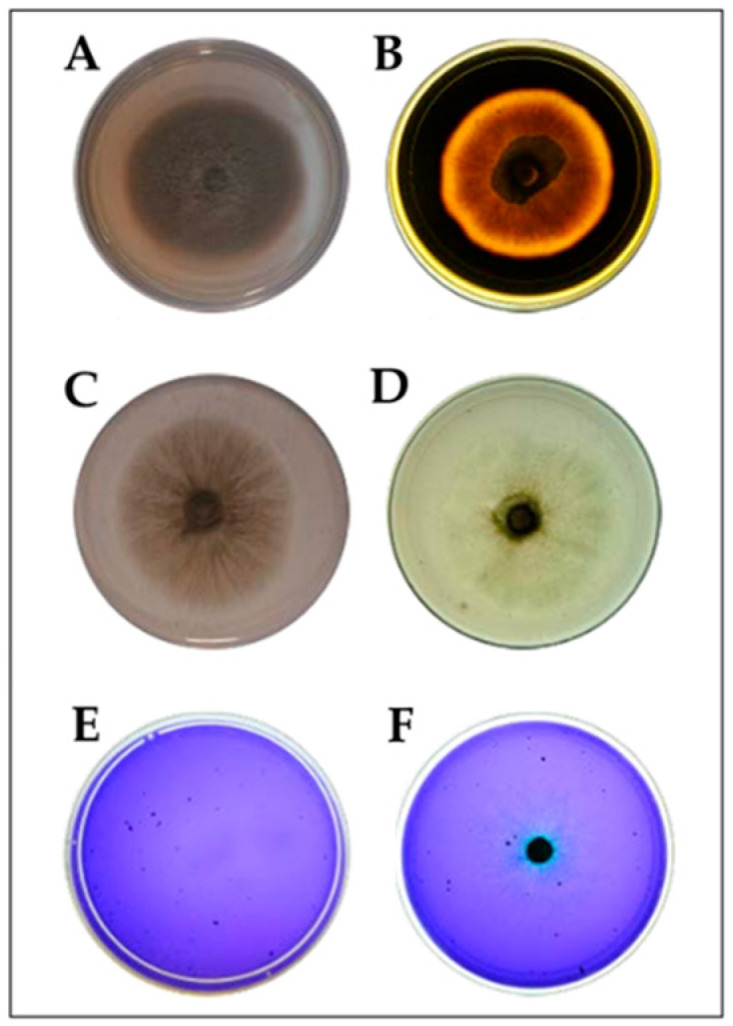
Qualitative assessment of the enzymatic activity of *Exserohilum rostratum*. (**A**) Growth of *E. rostratum* on medium containing starch as the sole carbon source; (**B**) detection of amylolytic activity; (**C**) growth of *E. rostratum* on medium containing carboxymethyl cellulose as the carbon source; (**D**) detection of cellulolytic activity; (**E**) growth of *E. rostratum* on medium supplemented with azure B dye; (**F**) detection of lignin peroxidase activity.

The production of extracellular enzymes (cellulases, amylases, and lignin peroxidases) to evaluate the qualitative potential to decompose recalcitrant organic compounds were registered in all the treatments with the studied fungus (Figure 2).

**Figure 3:**
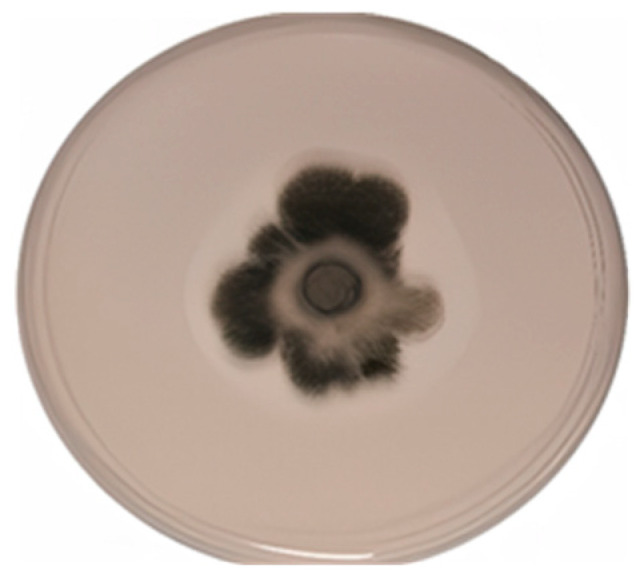
Phosphorous solubilization on Sundara’s agar plates by *E. rostratum*.

**Figure 4:**
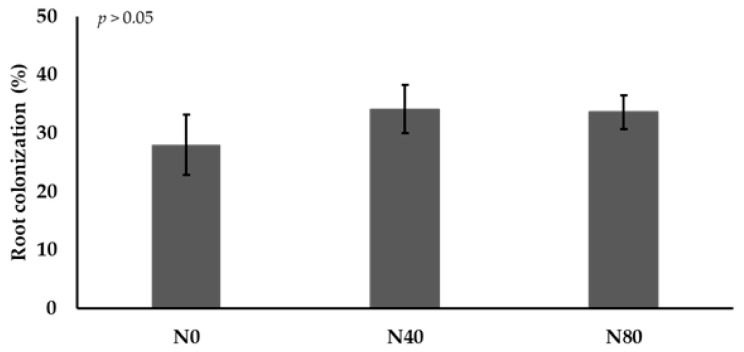
Percentage of roots colonized by DSE in studied plants growing under different salinity levels.

The fungus released acids (average control: pH 6.4, average fungus: pH 2.6) and possesses phosphate-solubilizing capacity (Figure 3). Root colonization is shown in Figure 4. The degree of *E. rostratum* colonization was around 30% and showed no effects of salinity.

### 3.2. Response of Chloris Gayana to Salinity and Fungus Inoculation

**Figure 5:**
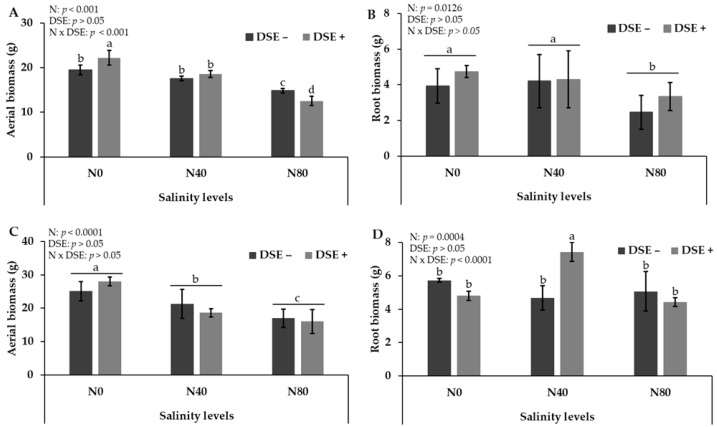
(**A**) Aerial biomass and (**B**) root biomass of *C. gayana* from the first experiment and (**C**) aerial biomass and (**D**) root biomass from the second experiment as affected by the interaction between salinity and DSE fungus inoculation. Different letters indicate significant differences (*p* < 0.05), according to Tukey’s multiple range test.

Aerial and roots biomass production of both experiments are shown in Figure 5.

Regarding aerial biomass in the first experiment, a significant interaction between salinity and DSE fungus inoculation was observed (*p* < 0.001). Inoculation with the fungus enhanced biomass production under non-saline conditions but did not affect biomass under moderate salinity (N40). Under severe salinity (N80), inoculated plants showed a reduction in aerial biomass compared to non-inoculated ones. In the second experiment, only the salinity level had a significant effect on aerial biomass (*p* < 0.0001). As salinity increased, *C. gayana* biomass decreased progressively, a trend consistent with that observed in the first experiment. On average, aerial biomass under the N80 treatments was approximately 60–70% of that observed under the N0 treatments. Root biomass remained stable when salinity increased to moderate levels (N40) but declined significantly under severe salinity (N80). No significant effect of DSE fungus inoculation was detected in the first experiment (*p* > 0.05) (Figure 5, Panel B).

In contrast, in the second experiment, a significant interaction between salinity and inoculation was observed (*p* < 0.0001). Inoculation with the DSE fungus did not alter root biomass under non-saline conditions (N0) or severe salinity (N80). However, under moderate salinity (N40), inoculated plants exhibited a significant increase in root biomass compared to non-inoculated plants.

Table 2 shows that foliar N, P, and crude protein (CP) contents increased significantly under high salinity (N80) in both control and inoculated treatments. Forage digestibility (FD) of *C. gayana* was strongly reduced under saline conditions compared to the control. Inoculation with the DSE fungus did not fully prevent this decrease; however, inoculated plants under moderate and high salinity showed slightly higher digestibility than their respective non-inoculated treatments.

### 3.3. Effect of the Fungi on Grass Tolerance to Salinity

Considering the extreme treatments (N0 without DSE and N80 with DSE) in both experiments, the STI values did not vary significantly, ranging from 0.29 to 0.44 (Supplementary Table S1). There were no significant differences in succulence (4.54 and 8.04%) among both inoculated and non-inoculated treatments. Also, the leaf greenness index, varying from 29.9 to 31.1 SPAD units, showed no significant differences among treatments.

**Figure 6:**
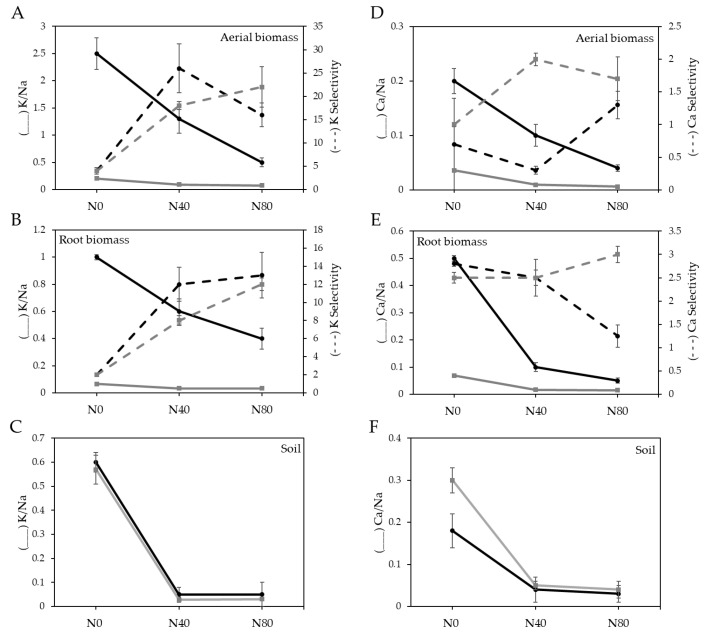
Interaction between salinity and fungus inoculation on ratios of K^+^/Na^+^ and Ca^2+^/Na^+^, as well as K^+^ and Ca^+^ selectivity in shoot and root biomass and in soil. Black lines represent treatments without DSE inoculation (DSE−), and grey lines represent treatments with DSE inoculation (DSE+).

Figure 6, Panel A shows the K/Na ratio in shoot biomass. This ratio decreased as salinity levels in the substrate increased. However, the K/Na ratio was consistently higher at all salinity levels in plants inoculated with the DSE fungus (*p* < 0.0001). Additionally, potassium selectivity was significantly greater (*p* < 0.001) in inoculated plants subjected to intermediate salinity levels (N40). Similar trends were observed for both the K/Na ratio and potassium selectivity in root biomass (Figure 6, Panel B). No significant differences (*p* > 0.05) were detected in the K/Na ratio in the substrate (Figure 6, Panel C).

Figure 6, Panels D and E show the Ca/Na ratio in the shoot and root biomass of *C. gayana*, respectively. As observed for the K/Na ratio, plants inoculated with *E. rostratum* exhibited significantly higher Ca/Na ratios across all salinity levels (*p* < 0.0001). Calcium selectivity in shoot biomass was significantly greater in inoculated plants exposed to intermediate salinity levels (N40) (*p* < 0.001). In root biomass, calcium selectivity was significantly greater in inoculated plants grown under non-saline conditions (N0) and significantly lower in roots of plants grown under severe salinity (N80) (Figure 6, Panel E). Finally, the Ca/Na ratio in the soil (Figure 6, Panel F) did not differ significantly between substrates with or without fungal inoculation (*p* > 0.05).

## 4. Discussion

Our results revealed differential effects of salinity on both the DSE fungus *Exserohilum rostratum* and the host grass *C. gayana*, as well as on their interaction under increasing salt concentrations. Contrary to what is typically observed in other studied fungi [8], sodium chloride did not negatively affect the mycelial biomass production of *E. rostratum*. This fungus demonstrated the ability to tolerate and accumulate high levels of Na without exhibiting marked growth inhibition. *E. rostratum* was also capable of producing extracellular enzymes, particularly lignin peroxidases, highlighting its potential to degrade recalcitrant organic compounds, an ability widely reported in other fungi [32]. Furthermore, the fungus significantly acidified the growth medium, which likely contributed to the mobilization of P from insoluble soil fractions as was previously reported by our group [30].

Root colonization by the DSE fungal strain was not hindered by salinity. Similar results were found in alfalfa (*Medicago sativa*) DSE-inoculated, grown under different sodium sulfate concentrations [3]. However, the absence of observable root colonization did not prevent the DSE fungus from positively influencing plant biomass. This supports earlier findings that DSE fungi can confer growth benefits even in the absence of visible root colonization [40], likely due to their enzymatic capacity to mineralize organic N and other compounds [41].

DSE inoculation frequently results in increased total biomass in grasses and other monocots [14,42]. Some studies report increases in both shoot and root biomass [43,44], while others observe enhancement primarily in roots [42]. In line with these reports, the response of *C. gayana* to salinity and inoculation with the DSE fungus *Exserohilum rostratum* revealed contrasting patterns for aerial and root biomass across experiments, highlighting the complexity of plant–fungus interactions under stress conditions.

For aerial biomass, the first experiment showed a significant interaction between salinity and fungal inoculation, where inoculation promoted growth under non-saline conditions but did not mitigate biomass loss under moderate salinity and even reduced it under severe salinity. In contrast, the second experiment showed no significant effect of inoculation, with salinity alone being the primary determinant of aerial biomass. These differences suggest that the positive effect of the fungus may depend on subtle environmental or physiological factors, such as nutrient availability or root–shoot allocation patterns, which can vary between experimental setups.

Root biomass responses also differed between experiments. In the first experiment, salinity reduced root biomass only at the highest salinity level, with no effect of inoculation detected. However, in the second experiment, a strong interaction between salinity and inoculation was observed: under moderate salinity, inoculated plants showed a significant increase in root biomass compared to non-inoculated plants. This suggests that *E. rostratum* may improve root development under certain stress thresholds, potentially through mechanisms such as enhanced water and nutrient uptake or modulation of osmotic adjustment as reported for other DSE fungi [41]. In all cases, and in accordance with others authors [45], shoot biomass was more negatively affected by salinity than root biomass.

The low values of the salt tolerance index (STI) observed in this study are consistent with the healthy appearance of the plants, which showed no visible signs of salinity-induced damage [21]. Moreover, Tao et al. (2021) [19] reported positive correlations between STI and various physiological parameters contributing to salt tolerance, including chlorophyll content, shoot water content, and the leaf K^+^/Na^+^ ratio, while negative correlations were found with the leaf Na^+^ content. Based on the STI values obtained here, *C. gayana* appears to lie at the threshold between salinity-tolerant and salinity-sensitive classifications, which partially contrasts with earlier reports that classified the species as salt-tolerant [16,17,18]. Leaf succulence was low and thus unlikely to play a significant role in salinity tolerance for this grass. Chlorophyll content was not negatively affected within the NaCl concentration range tested, and DSE inoculation had no effect on this parameter. This agrees with Daba et al. [16], who observed significant chlorophyll reduction only at salinity levels exceeding 10 dS m^−1^. The maintenance of chlorophyll levels may be attributed to restricted Na^+^ and Cl^−^ accumulation in the chloroplasts of photosynthetically active mesophyll cells, preserving photosynthetic function in *C. gayana* [26].

As commonly reported under saline conditions, the K^+^/Na^+^ and Ca^2+^/Na^+^ ratios decreased with increasing salinity in the substrate [25,26,27]. However, inoculated plants consistently showed higher K^+^/Na^+^ and Ca^2+^/Na^+^ ratios in both shoot and root biomass compared to non-inoculated plants, regardless of the salinity level. This suggests that *E. rostratum* inoculation may have promoted selective K^+^ and Ca^2+^ uptake or Na^+^ exclusion under stress conditions. While some studies have reported greater Na^+^ uptake in inoculated plants under salt stress [44], this was not the case here; instead, selectivity indices for K^+^ and Ca^2+^ were significantly higher in inoculated plants, particularly at moderate salinity levels (N40).

The ability to maintain a favorable K^+^/Na^+^ ratio is crucial for enzymatic activities, osmotic balance, and overall plant growth under saline conditions [19]. Although K^+^ selectivity remained high in inoculated plants, the overall biomass still decreased with increasing salinity, indicating that this compensatory mechanism was not sufficient to fully overcome the detrimental effects of salt stress.

Similarly, Ca^2+^/Na^+^ ratios were enhanced in inoculated plants across all salinity levels, and Ca^2+^ selectivity was highest under moderate salinity in shoots and under non-saline conditions in roots. Given that Ca^2+^ plays a key role in membrane stabilization and signaling under stress, this result suggests a potential protective effect conferred by fungal inoculation. However, as with K^+^, improved Ca^2+^ uptake did not completely prevent biomass reduction under severe salinity. Altogether, these findings indicate that *E. rostratum* positively influenced ionic homeostasis by promoting selective nutrient uptake, yet the fungal benefits were not sufficient to fully counteract high salinity stress. This contrasts with the findings of Ren et al. [3], who reported that DSE strains significantly improved alfalfa biomass and salt tolerance under increasing concentrations of Na_2_SO_4_. In their study, DSE inoculation led to increased catalase (CAT) activity, reduced Na^+^ accumulation in roots, and higher K^+^/Na^+^ ratios—mechanisms closely linked with physiological salt tolerance in plants. This discrepancy could be related to several factors: (i) differences in fungal strain identity and functional traits, (ii) host plant specificity in DSE–plant interactions, and (iii) the type of salt used (NaCl vs. Na_2_SO_4_) which differs in ionic composition and physiological effects on plants. Overall, our findings support the idea that while DSE fungi themselves may tolerate salinity, their ability to confer stress mitigation in plants is highly context-dependent.

Consistent with our findings, increased concentrations of N and P in plants inoculated with DSE fungi have been previously reported [14,44]. Similarly, Peterson et al. [43] found higher P concentrations in plant biomass and suggested that DSE fungi may facilitate internal P transport. In our case, differences in N and P concentrations may be partly explained by the well-known “dilution effect” observed in rapidly growing plants [46]. Crude protein content and forage digestibility are important indicators of the nutritional quality of grasses. The CP values observed in this study were comparable to those reported under field conditions in the study region [47]. Digestibility levels were similar to those observed under saline conditions in previous studies [16]. Although digestibility values decreased under salinity in all treatments, inoculation with the DSE fungus helped to maintain digestibility under moderate and high salinity (N40 and N80), showing slightly higher values than the respective non-inoculated treatments. While these differences were not statistically significant, they suggest that the fungal inoculation may mitigate the negative impact of salinity on forage quality.

## 5. Conclusions

The DSE fungus *Exserohilum rostratum* tolerated salinity without evident effects on its growth or root colonization ability. Functional traits such as extracellular enzyme production, substrate acidification, and phosphate solubilization were qualitatively observed under all tested conditions. The reduction in the aerial and root biomass of *Chloris gayana* due to salinity was within the range previously reported for this species, and all studied indices confirmed its tolerance to salinity. Inoculation with the DSE fungus significantly increased grass biomass under non-saline conditions, whereas its effects were limited under moderate salinity and absent at the highest salinity level. Although K^+^/Na^+^ and Ca^2+^/Na^+^ ratios decreased with increasing salinity, they remained higher in inoculated plants compared to controls. Unlike some other fungal symbionts, *E. rostratum* was not negatively affected by salinity but only conferred moderate benefits to the host plant under salt stress, with no significant advantages observed under severe saline conditions.

## Figures and Tables

**Table 1:** Tolerance of *Exserohilum rostratum* to NaCl concentrations in a liquid culture medium 12 days after the start of the test and Na accumulation in the mycelium, with its standard deviation. Different letters indicate significant differences at the different Na concentrations (*p* < 0.05) (*n* = 3).

Solution Na Concentration	Fungal Biomass (g)	Na Accumulation (mgNa/g Dry Mycelium)
Control	0.32 ± 0.07 b	0.020 ± 0.007 a
10 meq Na/L	0.42 ± 0.18 a	0.030 ± 0.009 a
100 meq Na/L	0.28 ± 0.09 b	0.400 ± 0.080 b

**Table 2:** Nitrogen, phosphorus, crude protein content, and digestibility of *C. gayana* as affected by salinity and fungus inoculation. Different letters indicate significant differences according to Tukey’s test (*p* < 0.05).

Treatment	N (%)	P (%)	Crude Protein (%)	Digestibility (%)
N0	0.67 ± 0.08 a	0.49 ± 0.05 a	4.18 ± 0.09 a	79.78 ± 3.12 a
N40	0.64 ± 0.05 a	0.58 ± 0.08 a	4.03 ± 0.08 a	61.74 ± 5.41 b
N80	0.98 ± 0.15 b	0.69 ± 0.07 b	6.12 ± 0.10 b	60.58 ± 4.00 b
N0 + DSE	0.77 ± 0.05 a	0.55 ± 0.04 a	4.81 ± 0.09 a	62.22 ± 3.17 b
N40 + DSE	0.72 ± 0.06 a	0.48 ± 0.07 a	4.70 ± 0.15 a	59.66 ± 4.57 b
N80 + DSE	0.92 ± 0.11 b	0.68 ± 0.12 b	5.75 ± 0.08 b	62.12 ± 5.21 b

## Data Availability

The data are contained in the article.

## Supplementary Materials

The following supporting information can be downloaded at https://www.mdpi.com/article/10.3390/plants14162537/s1.
