# Supplementary material for: Can the DSE Fungus Exserohilum rostratum Mitigate the Effect of Salinity on the Grass Chloris gayana?
Source: Plants (Basel). 2025 Aug 15;14(16):2537. doi: 10.3390/plants14162537 (PMC12389101; doi:10.3390/plants14162537)
Supplement: Supplementary file 1 [file plants-14-02537-s001.zip › plants-3762981-supplementary.pdf]

**Supplementary Table S1.** Salt Tolerance Index (STI) values (control vs. saline stress) of non-inoculated and *Exserohilum rostratum*-inoculated plants subjected to salinity stress.

| Experiment | Non-inoculated plants | Inoculated plants |
|---|---|---|
| **1** | 0.29 ± 0.06 | 0.43 ± 0.18 |
| **2** | 0.35 ± 0,05 | 0.44 ± 0.07 |
